# Minimum inhibitory concentration of vancomycin to methicillin resistant *Staphylococcus aureus* isolated from different clinical samples at a tertiary care hospital in Nepal

**DOI:** 10.1186/s13756-016-0126-3

**Published:** 2016-07-21

**Authors:** Arjun Ojha Kshetry, Narayan Dutt Pant, Raju Bhandari, Sabita Khatri, Krishma Laxmi Shrestha, Shambhu Kumar Upadhaya, Asia Poudel, Binod Lekhak, Bijendra R. Raghubanshi

**Affiliations:** Department of Microbiology, Goldengate International College, Battisputali, Kathmandu, Nepal; Department of Microbiology, Grande International Hospital, Dhapasi, Kathmandu, Nepal; Department of Microbiology, KIST Medical College and Teaching Hospital, Lalitpur, Nepal

**Keywords:** *S. aureus*, MRSA, VRSA, VISA, Nepal

## Abstract

**Background:**

Methicillin resistant *Staphylococcus aureus* (MRSA) has evolved as a serious threat to public health. It has capability to cause infections not only in health care settings but also in community. Due to the multidrug resistance shown by MRSA, there are limited treatment options for the infections caused by this superbug. Vancomycin is used as the drug of choice for the treatment of infections caused by MRSA. Different studies from all around the world have documented the emergence of strains of *S. aureus* those are intermediate sensitive or resistant to vancomycin. And recently, there have been reports of reduced susceptibility of MRSA to vancomycin, from Nepal also. So the main purpose of this study was to determine the minimum inhibitory concentration (MIC) of vancomycin to methicillin resistant *S. aureus* isolated from different clinical specimens.

**Methods:**

Total 125 strains of *S. aureus* isolated from different clinical samples at KIST Medical College and Teaching Hospital, Lalitpur, Nepal from Nov 2012 to June 2013, were subjected to MRSA detection by cefoxitin disc diffusion method. The minimum inhibitory concentrations of vancomycin to confirmed MRSA strains were determined by agar dilution method. Yellow colored colonies in mannitol salt agar, which were gram positive cocci, catalase positive and coagulase positive were confirmed to be *S. aureus*.

**Results:**

Among, total 125 *S. aureus* strains isolated; 47(37.6%) were MRSA. Minimum inhibitory concentrations of vancomycin to the strains of MRSA ranged from 0.125 μg/ml to 1 μg/ml.

**Conclusion:**

From our findings we concluded that the rate of isolation of MRSA among all the strains of *S. aureus* isolated from clinical samples was very high. However, none of the MRSA strains were found to be vancomycin intermediate-sensitive or vancomycin-resistant.

## Background

*Staphylococcus aureus* is present as the normal flora of humans; on skin and anterior nares and up to two-third of the population is colonized by it [1]*.* It is a pathogenic bacterium; which may be responsible for causing a broad spectrum of diseases in hospital as well as the community and can develop drug resistance to commonly used antibiotics in short period of time [2]. Most importantly, *S. aureus* causes skin and soft tissue infections but it can also cause fatal diseases like endovascular infections and sepsis [2–4]. Colonization of the anterior nares in humans is one of the most important risk factors for infection by *S. aureus* [5]. Despite the development of improved surgical techniques and antibiotic prophylaxis, the infections caused by *S. aureus* present as a serious public health problems [6]. Virtually no strains of *S. aureus* are known to be intrinsically resistant to any antibiotics developed till date [7].

Since 1960s, methicillin-resistant *S. aureus* (MRSA) has established itself as one of the commonest causes of nosocomial infections and its dissemination to the community has created a more dreadful situation [8, 9]. For the treatment of MRSA; vancomycin is used as drug of choice [10]. However, there are increasing numbers of reports on emergence of vancomycin intermediate-sensitive *S. aureus* (VISA) and vancomycin-resistant *S. aureus* (VRSA) [11]. In addition, many researchers have reported the higher rates of likelihood of treatment failure leading to higher rates of mortality, due to the infection caused by MRSA having MIC of vancomycin at the upper end of susceptible range [12]. The higher MIC of vancomycin to MRSA even in the susceptible range correlates with the drug resistance to many different classes of the antibiotics [11]. Till date no VRSA has been reported from Nepal but the increasing trends of decreased susceptibility of the strains of MRSA to vancomycin reported from Nepal, indicate the need of more researches in the field [13]. In Nepal, only limited data are available on the susceptibility of MRSA to reserve drug, vancomycin. So, in this study we determined the minimum inhibitory concentration of vancomycin to methicillin resistant *Staphylococcus aureus* isolated from different clinical specimens.

## Methods

The study was conducted at KIST Medical College and Teaching Hospital, Lalitpur, Nepal from Nov 2012 to June 2013, using the total of 125 strains of *Staphylococcus aureus* isolated from different clinical samples. Yellow colored colonies on mannitol salt agar, which were gram positive cocci, catalase positive and coagulase positive were confirmed to be *S. aureus.* Antimicrobial susceptibility testing of *S. aureus* to penicillin and vancomycin was performed by modified Kirby-Bauer disc diffusion technique, following clinical and laboratory standards institute (CLSI) guidelines [14].

### Detection of methicillin resistant *S. aureus*

Detection of MRSA was performed by using cefoxitin disc (30 μg). For this lawn culture was performed on Mueller-Hinton agar plate, using the broth culture of *S. aureus* with turbidity adjusted to 0.5 McFarland standard. Then a cefoxitin disc (30 μg) was kept on the lawn culture after it had been left to dry for about 5 min. Finally, the agar plate was incubated aerobically at 35 ^o^C for 18 h. The strains showing diameter of zone of inhibition of ≤ 21 mm were considered as methicillin resistant *S. aureus* (MRSA) while those with diameter of zone of inhibition of ≥ 22 mm were identified as methicillin susceptible *S. aureus* (MSSA) [14].

### Screening of β-lactamase producers among *S. aureus*

Penicillin disc zone-edge test was used to identify the β-lactamase producing strains of *S. aureus*. For this penicillin G (10 units) disc was used. The strains showing sharp zone edge were screened to be β-lactamase producers while those indicating fuzzy zone edge were considered to be β-Lactamase non-producers [14].

### Determination of minimum inhibitory concentration of vancomycin to MRSA

The minimum inhibitory concentrations of vancomycin to the strains of MRSA were determined by agar dilution method as suggested by Andrews [15] following CLSI guidelines [14]. The different dilutions of vancomycin used were 0.0625 μg/ml to 64 μg/ml.

### Quality control

For quality control *S. aureus* (ATCC 25923) was used.

### Data analysis

The data obtained were analysed by using statistical package for social sciences (SPSS) version 16. Chi-square test was used and p-value < 0.05 was taken as significant.

## Results

Among a total of 125 *S. aureus* isolates, 47(37.6%) were methicillin resistant (MRSA).

### Distribution of *S. aureus* on the basis of different clinical specimens

Most of the strains of *S. aureus* were isolated from pus (66.4%) followed by blood (13.6%) and urine (8.8%) (Table 1).

**Table 1:** Distribution of *S. aureus* on the basis of different clinical specimens

Clinical Specimens	*S. aureus*		Total (%)
	MRSA	MSSA	
Pus	27	56	83(66.4)
Blood	10	7	17(13.6)
CAPD fluid	1	0	1(0.8)
Sputum	4	6	10(8)
Urine	3	8	11(8.8)
Sub-Clavian Tip	2	0	2(1.6)
Breast Milk	0	1	1(0.8)
Total	47	78	125

### Distribution of MRSA on the basis of different parameters

All the patients were categorized into two categories (<18 years of age as pediatric and the people >19 years of age as adult). In our study, 29 strains of MRSA were isolated from adults while 18 isolates of MRSA were isolated from pediatric patients and the difference was statistically insignificant (*p* > 0.05). Similarly, 30 MRSA strains were isolated from male and 17 MRSA strains were isolated from female. The distribution of MRSA among the patients on the basis of sex was found to be statistically significant (*p* < 0.05). Further, 28 isolates of MRSA were isolated from in-patients department while 19 MRSA strains were isolated from out-patient department and the difference was statistically insignificant (*p* > 0.05) (Table 2).

**Table 2:** Distribution of MRSA on the basis of different parameters

Parameters		MSSA	MRSA	P-value
Age	Pediatric	19	18	0.098
	Adult	59	29	
Gender	Male	30	30	0.006
	Female	48	17	
Department	OPD	44	19	0.083
	IPD	34	28	

### Rate of β-lactamase production among penicillin resistant *S. aureus*

All the 125 *S. aureus* isolates were resistant to penicillin by disc diffusion technique. However, only 69(55.2%) of the isolates were found to be resistant to penicillin due to β-lactamase production.

### Distribution of β-lactamase producer penicillin resistant *S. aureus* on the basis of different parameters

The distribution of β-lactamase producer penicillin resistant *S. aureus* on the basis of different parameters like age, sex and departments was found to be statistically insignificant (*P* > 0.05) (Table 3).

**Table 3:** Distribution of β–lactamase producing and non-producing *S. aureus*

Parameters		β -lactamase production		P-value
		+	-	
Age	Pediatric	19	18	0.575
	Adult	50	38	
Gender	Male	28	32	0.065
	Female	41	24	
Department	OPD	37	26	0.424
	IPD	32	30	

Note: + = Positive, - = Negative

### Minimum inhibitory concentrations (MIC) of vancomycin to MRSA isolates

Most of the strains of MRSA showed resistance toward vancomycin on performing antimicrobial susceptibility testing by disc diffusion method using vancomycin disc. However, the determination of minimum inhibitory concentrations of vancomycin to MRSA isolates showed that all the strains of MRSA were susceptible to vancomycin with MIC ranging from 0.125 μg/ml to 1 μg/ml. The numbers of the MRSA isolates having different MIC of vancomycin and showing different sizes of zone of inhibition for vancomycin disc are given in Table 4.

**Table 4:** Minimum inhibitory concentrations of vancomycin to MRSA isolates versus zones of inhibition of MRSA for vancomycin

MIC of vancomycin (μg/ml)	Zones of inhibition for vancomycin (mm)										
	6	7	8	9	10	11	12	13	14	15	16
0.125						1		1	1		1
0.25				1	1	1	1	1	1		
0.5	2		3	1	5	1	4	4	3	1	4
1	1	1		1	1	1	2		2		

## Discussion

*S. aureus* is a flexible pathogen, which may be responsible for causing community acquired as well as nosocomial infections [1]. Despite the years of efforts to develop the new antibiotics for the eradication of MRSA, it has established itself as the commonest cause of skin and soft tissue infections [16, 17]. The prevalence of MRSA in our study was 47/125(37.6%), which was consistent with the results reported by Sanjana et al. (39.6%) [18] and Juayang et al. (40.6%) [19]. However, lower prevalence of MRSA in comparison to our study were reported by Subedi et al. (15.4%) [20], Baral et al. (26%) [21], Pandey et al. (26.12%) [22] and Kumari et al. (26.14%) [23]. While higher prevalence of MRSA were reported by Arora et al. (46%) [24], Dibah et al. (46.3%) [25], Khanal et al. (68%) [26] and Tiwari et al., (69.1%) [27]. The difference in the prevalence of MRSA among different studies may be due to difference in the location and time period of the study. The prevalence of MRSA may differ from one hospital to another hospital, depending upon the types of the patients it receives, hygienic condition of the hospital and the health care workers. If the hospital is a referral center then the prevalence of the MRSA among the patients may be very high, as the chance of getting antimicrobial therapy before reaching the referral center is very high and due to selective pressure the bacteria may acquire drug resistance. Healthcare workers may be not only the important source of transmission of MRSA to patients or among patients but also to the community [28]. Strict implementation of hand hygiene and decolonization of the MRSA carriers will be helpful to control the transmission of MRSA [11]. In addition, maintaining good (environmental as well as personal) hygiene in the hospital, among healthcare workers and patients will be more beneficial [11]. Due to the availability of limited treatment options for infections caused by MRSA, the treatment of such infections is often difficult leading to prolonged hospital stay and longer course along with higher cost of treatment sometimes leading to treatment failure resulting into fatal outcome [28]. Further, the higher rate of isolation of MRSA from clinical specimens of patients suggests the more attention to be given for infection control and surveillance, which may increase the overall infection control cost in the hospital [29].

In our study no strains of MRSA were found to be vancomycin resistant or vancomycin intermediate sensitive and the minimum inhibitory concentrations of vancomycin to the strains of MRSA ranged from 0.125 μg/ml to 1 μg/ml. As in our study, in a research from Nepal; Amatya et al. also did not note any strains of MRSA to be VISA or VRSA but higher MIC (i.e., 0.5 μg/ml to 2 μg/ml) of vancomycin to MRSA (in comparison to our study) was reported [11]. In contrast, in another study from Nepal; Pahadi et al. reported the four isolates of the MRSA to be VISA, with MIC of vancomycin to all the MRSA, ranging from 0.5 μg/ml to 4 μg/ml [13]. The discrepancy seen in results of different studies conducted in Nepal, may be because of the involvement of the patients with previous history of exposure to vancomycin in some studies. No VRSA has yet been reported from Nepal [13]. Decreased susceptibility of *S. aureus* to vancomycin was reported first from Japan in 1997 [30]. And the first strain of VRSA was isolated in 2002 from Michigan, USA [31]. Since then the VISA and VRSA have been reported frequently by many researchers [32–36].

The mechanism behind the resistance of *Staphylococcus aureus* to vancomycin may be the thickening of cell wall [37]. In addition; prior exposure to vancomycin increases the chances of the isolation of the strains of *Staphylococcus aureus* with reduced susceptibility [11] and the reason for emergence of VRSA/VISA may be the selective pressure due to the haphazard use of the antibiotic (vancomycin) [11]. The morbidity and mortality due to infection caused by VRSA are very high because of limited treatment options available [33]. At present when the infections due to MRSA have become a serious public health concern; the development and rapid spread of resistance of *S. aureus* to the reserve drug (vancomycin) is very fearsome and immediate actions should to be taken by the responsible authorities to halt it [11].

Due to haphazard use of antibiotics, there is increasing trend of development of drug resistance among the bacteria and the condition is more critical in poorer countries [38, 39]. To prevent the situation of the drug resistance from worsening; the use of antibiotic for the treatment of the patients should be based on culture and sensitivity report [40].

### Limitations of the study

Inability to use the molecular methods in our study is the main limitation of our research. Further, we could not differentiate between community acquired MRSA and hospital acquired MRSA. In addition, we were unable to include more samples in our study. National level study involving large numbers of samples would have generated more significant results and the actual situation of the MIC of vancomycin to MRSA, in Nepal.

## Conclusion

In our study the rate of isolation of MRSA among all the strains of *S. aureus* was very high. But none of the MRSA strains were found to be vancomycin intermediate-sensitive or vancomycin-resistant. The MIC of vancomycin for MRSA in our study was found to be lower in comparison to similar other studies conducted in Nepal.

## Abbreviations

ATCC, American Type Culture Collection; CAPD, continuous ambulatory peritoneal dialysis; CLSI, Clinical and laboratory Standards Institute; IPD, In patient Department; MIC, minimum inhibitory concentration; MRSA, methicillin resistant *S. aureus*; MSSA, methicillin susceptible *S. aureus*; OPD, Out Patient Department; SPSS, Statistical Package for the Social Sciences; USA, United States of America; VISA, vancomycin intermediate sensitive *S. aureus*; VRSA, vancomycin resistant *S. aureus*
